# Rheumatoid factor positivity in ANCA-negative eosinophilic granulomatosis with polyangiitis: cardiac mimicry, mononeuritis multiplex, and long-term outcome in a case-based systematic review

**DOI:** 10.1007/s00296-026-06334-7

**Published:** 2026-11-10

**Authors:** Ali D. Alghaithi, Carol Saryeddine, Rashid D. Alghaithi, Gehad Elghazali, Khalid A. Alnaqbi

**Affiliations:** 1 Department of Medicine, Sheikh Tahnoon bin Mohammed Medical City, SEHA/PureHealth, Al Ain, UAE; 2 Faculty of Medicine, American University of Beirut, Beirut, Lebanon; 3 School of Medicine, Royal College of Surgeons in Ireland (RCSI), University of Medicine and Health Sciences, Dublin, Ireland; 4 Sheikh Khalifa Medical City, PureLab, Abu Dhabi, UAE; 5 Division of Rheumatology, Sheikh Tahnoon bin Mohammed Medical City, SEHA/PureHealth, Al Ain, UAE; 6 College of Medicine, RAK Medical and Health Sciences University, Ras Al Khaimah, UAE; 7 Internal Medicine Department, College of Medicine and Health Sciences, UAE University, P. O. Box 1551, Al Ain, UAE

**Keywords:** Eosinophilic granulomatosis with polyangiitis, Anti-neutrophil cytoplasmic antibody-associated vasculitis, Mononeuropathies, Myocardial infarction, Rheumatoid factor, Eosinophilia

## Abstract

Eosinophilic granulomatosis with polyangiitis (EGPA) is a rare small- to medium-vessel vasculitis characterized by asthma, eosinophilia, and multisystem involvement. Diagnosis is challenging in ANCA-negative disease, particularly when misleading serology is present. We describe a 42-year-old woman with long-standing asthma and a rheumatoid factor (RF) of 322 IU/mL, who initially presented with myocardial infarction–like cardiac involvement with non-obstructive coronary arteries. Over the subsequent seven months, progressive eosinophilia, chronic rhinosinusitis with nasal polyposis, and mononeuritis multiplex emerged. ANCA-negative EGPA was diagnosed after connective tissue disease, infection, malignancy, and other causes of hypereosinophilia were excluded. She was treated with prednisolone and azathioprine and subsequently remained off immunosuppressive therapy for more than nine years without reported relapse. On later clinical reassessment, she remained stable apart from mild residual neuropathic symptoms. We also conducted a case-based review following the CABARET standards, searching PubMed/MEDLINE, Scopus, Embase, and Web of Science up to 15 August 2026. We identified 38 published cases of ANCA-negative, RF-positive EGPA, 36 of them without pre-existing rheumatoid arthritis. In these 36, the median age was 53 years and 69% were female. Peripheral neuropathy was reported in 72% and cardiac involvement in 47%; with both occurring in 25%; two patients died of cardiac causes. Among 12 cases with sufficient data to standardize RF relative to the assay upper limit of normal, 75% had RF levels > 3 × ULN. Reporting was often incomplete: both MPO- and PR3-ANCA were documented in only 42% of cases and anti-CCP in 11%. No included published case reported prolonged treatment-free remission, although follow-up and treatment status were incompletely reported in several cases. Based on these gaps, the authors propose a reporting checklist for future EGPA case reports. RF positivity, even at high titer, should not dissuade clinicians from considering EGPA when asthma, eosinophilia, ENT disease, neuropathy, or other compatible manifestations are present.

## Key points

- ANCA negativity does not exclude EGPA; among the included published ANCA-negative, RF-positive cases, neuropathy was reported in 72% and cardiac involvement in 47%.
- Isolated rheumatoid factor positivity is non-specific and may act as a diagnostic distraction.
- RF positivity, even at high titers, should not dissuade clinicians from considering EGPA when asthma, eosinophilia, and other compatible manifestations are present.
- Prolonged treatment-free remission may occur in EGPA, although long-term outcomes remain heterogeneous and require further characterization.

## Introduction

ANCA-associated vasculitides (AAV) are rare small- to medium-vessel systemic diseases comprising granulomatosis with polyangiitis, microscopic polyangiitis, and eosinophilic granulomatosis with polyangiitis (EGPA). EGPA is characterized by eosinophilic inflammation and heterogeneous multisystem involvement, most commonly including asthma (85.5%), ear, nose, and throat manifestations (75.8%), pulmonary involvement (74.2%), neurological manifestations (51.5%), and cardiac involvement (27.7%) [1]. In the Arabian Gulf countries, EGPA accounts for approximately 12% to 26.5% of all AAV cases [2–4].

The heterogeneous and evolving clinical manifestations of EGPA contribute to significant diagnostic complexity. Individual features often emerge at different time points and may initially appear unrelated, delaying recognition of the underlying vasculitic process. This challenge is particularly evident in ANCA-negative disease, in which early manifestations such as asthma, chronic rhinosinusitis, and eosinophilia are frequently attributed to allergic or infectious disorders, while eosinophilic cardiac involvement may remain clinically subtle or unrecognized [5].

Rheumatoid factor (RF) is an autoantibody directed against the Fc portion of IgG that lacks disease specificity. Although detected in approximately 70% to 90% of patients with rheumatoid arthritis (RA), RF may also be present in other autoimmune diseases, including Sjögren disease and systemic lupus erythematosus, as well as in systemic vasculitides, chronic infections, malignancies, and even healthy older adults [6]. Consequently, isolated RF positivity should always be interpreted within the overall clinical context and should not be considered diagnostic of RA.

We report a diagnostically challenging case of ANCA-negative EGPA in which marked RF positivity preceded the development of multisystem disease, including myocardial infarction with non-obstructive coronary arteries (MINOCA) and mononeuritis multiplex. The patient subsequently remained off immunosuppressive therapy for more than nine years without reported relapse, with later clinical reassessment confirming continued stability. This case highlights the importance of integrating seemingly unrelated clinical features to facilitate earlier recognition of EGPA despite the absence of ANCA positivity.

## Case description

A 42-year-old Omani premenopausal woman with a 15-year history of asthma was admitted to internal medicine on 29 November 2013 with a two-week history of chest pain, chronic productive cough, unintentional weight loss of 6 kg, night sweats, and chills. She was a lifelong non-smoker and did not consume alcohol. She had no prior history of hypertension, diabetes mellitus, hyperlipidemia, or known cardiovascular (CV) disease. Family history was negative for autoimmune disease, vasculitis, malignancy, or premature coronary artery disease. She had experienced recurrent, poorly controlled asthma over the preceding year with multiple emergency department visits. She reported no recent travel, new medication use, or illicit drug use.

Initial investigations showed a low hemoglobin level of 116 g/L (normal 131–172) with microcytic hypochromic anemia, positive RF at 322 IU/mL (positive if  > 19 IU/mL) and a high C-reactive protein (CRP) level of 55.4 mg/L (normal < 7.5 mg/L). Erythrocyte sedimentation rate (ESR) was not measured. She had normal white blood cells (WBC) count of 7.0 × 10^9^/L, an eosinophil count of 0.1% (normal range 1.0–6.0%), creatinine was normal at 45.4 µmol/L (normal range 35–88). PR3-ANCA and MPO-ANCA were negative by Enzyme-Linked Immunosorbent Assay (ELISA). Infectious evaluation revealed a positive QuantiFERON-TB test with repeatedly negative sputum acid-fast bacilli. Serum tumor markers, including CA 125, CA 19 − 9, and CA 15 − 3, were normal. Contrast-enhanced CT of the chest demonstrated bilateral upper lobe fibrotic changes, scattered micronodules, tree-in-bud opacities, and right lower lobe consolidation. CT abdomen and pelvis with contrast was normal. Transthoracic echocardiography (TTE) demonstrated mild inferobasal hypokinesia with preserved left ventricular systolic function (55%).

**Fig. 1:**
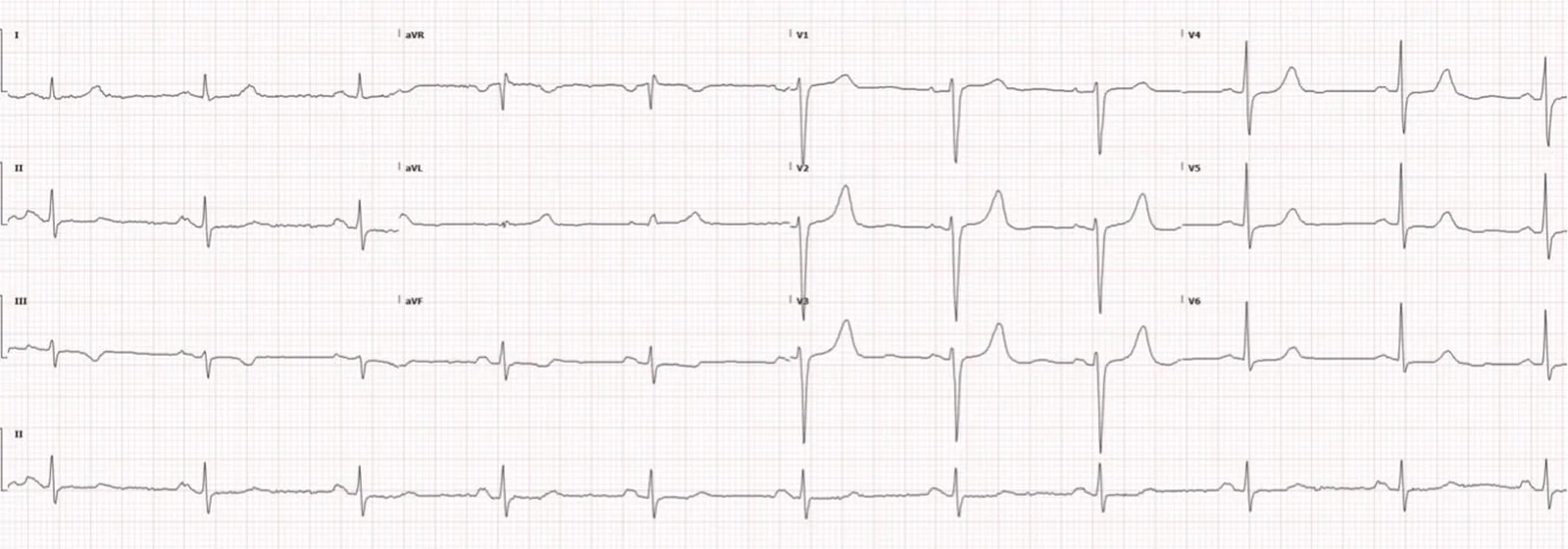
ECG showing normal sinus rhythm, normal axis, and nonspecific inferior T-wave abnormalities, most prominent in inferior leads (II, III, aVF)

During admission, cardiology was involved due to persistent chest pain associated with elevated troponin I levels. Further history revealed that she had been evaluated in Oman one month earlier for suspected non-ST-elevation myocardial infarction (NSTEMI), and elective percutaneous coronary intervention had been planned before her presentation to our hospital. Her ECG is shown in Fig. 1. She was discharged on dual antiplatelet therapy (aspirin and clopidogrel), bisoprolol, high-intensity atorvastatin, and inhaled asthma therapy (fluticasone/salmeterol with salbutamol as required). Outpatient assessment for myocardial ischemia was arranged, and she also commenced nine months of latent tuberculosis treatment with isoniazid and pyridoxine. The markedly elevated RF prompted rheumatology referral.

In January 2014, she was evaluated in the rheumatology clinic. She had no clinical features suggestive of connective tissue disease (CTD), infective endocarditis, mucocutaneous, gastrointestinal, neurological, musculoskeletal, or renal involvement. Specifically, she reported no headache or seizures. Laboratory investigations showed a high CRP level of 80.1 mg/L. Extensive autoimmune and infectious screening, including antinuclear antibodies (ANA), extractable nuclear antigens, anti-cyclic citrullinated peptide (CCP) antibodies, complement levels, cryoglobulins, hepatitis B and C serology, syphilis, and HIV testing, was negative. Serum protein electrophoresis, creatinine, urinalysis, and urine microscopy were normal. The isolated elevation in rheumatoid factor was considered non-specific.

On 12 February 2014, myocardial perfusion imaging using technetium-99 m sestamibi single-photon emission computed tomography (SPECT) demonstrated mild-to-moderate predominantly reversible anteroseptal and inferolateral perfusion defects with an estimated left ventricular ejection fraction of approximately 45%, raising concern for myocardial ischemia or inflammatory cardiac involvement. Coronary CT angiography was not performed because of contrast allergy.

On 29 June 2014, she was admitted under neurology for progressive right lower limb pain with paresthesia involving the knee and foot that had developed over several months, accompanied by perceived muscle wasting of the right leg and increasing difficulty with toe walking. Neurological examination demonstrated right distal lower limb weakness (extensor hallucis longus weakness and ankle dorsiflexion grade 4/5), reduced right ankle reflex, and asymmetric sensory loss, consistent with an evolving asymmetric peripheral neuropathy. The clinical impression was progressive mononeuritis multiplex, with concern for an underlying systemic inflammatory, vasculitic, or infiltrative process. Nerve conduction studies demonstrated a right tibial axonal neuropathy and early right sural nerve damage. Peripheral WBC (6.7 × 10^9^/L), CRP level (5.9 mg/L), and ESR (11 mm/hr) were normal. Antiphospholipid antibody (APLA) testing showed transient positivity for anticardiolipin IgG and β2-glycoprotein I IgG, whereas IgM antibodies and lupus anticoagulant were negative; repeat testing in November 2014 was negative. Urinalysis, urine microscopy, and metabolic investigations (HbA1c, vitamin B12, serum protein electrophoresis) were unremarkable. Serial hematological monitoring demonstrated progressive eosinophilia from 0.1% in November 2013 to 8.7% (0.8 × 10^9^/L, normal range ≤ 0.5 × 10^9^/L) in January 2014, 32.3% (2.3 × 10^9^/L) in March 2014, and 30.3% (2.0 × 10^9^/L) in June 2014. Concurrently, serum IgE increased from 374 IU/mL in January 2014 to 1040 IU/mL in June 2014. Aspergillus fumigatus–specific IgE was undetectable. CT sinus with contrast confirmed extensive chronic rhinosinusitis with nasal polyposis. The patient declined a nerve biopsy.

Following multidisciplinary discussion involving rheumatology, pulmonology, and neurology, a diagnosis of ANCA-negative EGPA was established based on adult-onset asthma, chronic rhinosinusitis with nasal polyposis, NSTEMI in the absence of traditional CV risk factors, progressive eosinophilia, progressively elevated IgE, systemic symptoms, and evolving mononeuritis multiplex. She fulfilled the 2022 American College of Rheumatology / EULAR classification criteria for EGPA, with a score of 12 (a score of ≥ 6 is required) [7]. The Five-Factor Score was 0. HLA typing was performed using low-to-intermediate sequence-specific oligonucleotide (SSO) typing on a Luminex platform and demonstrated homozygosity for the HLA-DRB1*03:01-DQA1*05:01-DQB1*02:01 (HLA-DR3-DQ2 haplotype).

Prednisolone 45 mg daily (1 mg/kg; body weight 45 kg) was started with a planned gradual taper over one year. Because of concerns regarding potential adverse effects, the patient declined treatment escalation to cyclophosphamide or rituximab. Following corticosteroid initiation, her neurological symptoms gradually improved, respiratory disease stabilized, and no further cardiac events occurred.

On 16 November 2014, azathioprine 100 mg daily (2 mg/kg) was introduced as steroid-sparing maintenance therapy. The medication was continued until May 2016, after which the dose was reduced to 50 mg daily for one year before the patient discontinued treatment without medical advice. Neuropathic symptoms were managed symptomatically with pregabalin. Overall, treatment was well tolerated, with no reported adverse events.

Coronary angiography performed in September 2014 demonstrated normal epicardial coronary arteries, excluding obstructive coronary artery disease. Interpreted together with the earlier TTE showing inferobasal hypokinesia and the SPECT demonstrating reversible perfusion defects with mildly reduced left ventricular ejection fraction, the overall cardiac presentation was most consistent with MINOCA, with inflammatory, microvascular, or vasospastic mechanisms considered the most likely explanation in the context of EGPA. Cardiology follow-up in 2016 and 2017 confirmed sustained clinical stability with no ischemic symptoms; rheumatology documented stable disease activity, and pulmonology follow-up demonstrated stable moderate obstructive airway disease.

The patient was reviewed in the rheumatology clinic on 17 March 2019 and was clinically stable. She was subsequently lost to follow-up and re-presented on 30 September 2025. She reported excellent functional status with only mild intermittent distal lower-limb sensory symptoms and no systemic complaints, and had remained off immunosuppressive therapy since 2017 without relapse. Investigations showed mild leukopenia (3.6 × 10⁹/L) with mild eosinophilia (9.2%, 0.33 × 10^9^/L), normal routine blood tests (renal and hepatic function, ESR 15 mm/hr, and CRP 3.3 mg/L). Autoimmune serology, including rheumatoid factor, anti-CCP, ANA, PR3-ANCA and MPO-ANCA by chemiluminescence immunoassay (CLIA), was negative. Chest xrays were normal. TTE revealed preserved biventricular function without evidence of cardiomyopathy or pulmonary hypertension. Repeat nerve conduction studies in September 2025 showed only mild chronic residual axonal changes without evidence of active or progressive neuropathy. A follow-up rheumatology telemedicine visit in October 2026 revealed a stable condition. Table 1 depicts the chronological clinical events of our patient.

**Table 1:** Chronological clinical course by disease phase

Date	Clinical findings & events	Key investigations & results	Management / outcome
**Phase 1—Initial presentation (October–November 2013)**			
October 2013	Chest pain at age 42; diagnosed as NSTEMI	Elective PCI planned.	–
November 2013	2-week chest pain, chronic productive cough, 6 kg weight loss, night sweats, chills. 15-year asthma, poorly controlled; lifelong non-smoker.	Troponin I ↑; eosinophils 0.1%. RF 322 IU/mL; CRP 55.4 mg/L. Negative PR3-ANCA and MPO-ANCA. CT of the chest: bilateral upper lobe fibrotic changes, scattered micronodules, tree-in-bud opacities, and right lower lobe consolidation. TTE: mild inferobasal hypokinesia with preserved left ventricular systolic function (55%).	Dual antiplatelet, bisoprolol, atorvastatin, inhaled fluticasone/salmeterol, salbutamol. Started 9-month latent-TB therapy (isoniazid + pyridoxine); rheumatology referral for high RF.
**Phase 2—Diagnostic evaluation (January–February 2014)**			
January 2014	Rheumatology clinic: No features of connective tissue disease, malignancy, infection, or endocarditis.	CRP 80.1 mg/L; eosinophilia 8.7% (0.8 × 10^9^/L).	Clinical monitoring; no immunosuppression yet.
February 2014	Concern for myocardial ischemia or inflammatory cardiac involvement.	Tc-99 m sestamibi SPECT: mild–moderate, predominantly reversible anteroseptal and inferolateral perfusion defects; LVEF ~ 45%. CT coronary angiography deferred (contrast allergy).	–
**Phase 3—EGPA diagnosis (June 2014)**			
June 2014	Progressive right lower-limb pain and paresthesia over several months; difficulty toe-walking. Exam: distal weakness (EHL, ankle dorsiflexion 4/5), reduced ankle reflex, asymmetric sensory loss → mononeuritis multiplex.	NCS: right tibial axonal neuropathy and early right sural (later left superficial peroneal) → vasculitic pattern. Eosinophils 32.3%, 2.3 × 10^9^/L (March 2014) → 30.3%, 2.0 × 10^9^/L (June 2014). IgE 374 → 1040 IU/mL.	ANCA-negative EGPA diagnosed (multidisciplinary).
**Phase 4—Induction & maintenance therapy (June–November 2014)**			
June 2014	Treatment initiated for cardiac and neurological involvement.	–	Prednisolone 45 mg/day (1 mg/kg), tapered over 1 year. Patient declined nerve biopsy and escalation to other immunosuppressive therapies.
September 2014	Cardiac mechanism clarified.	Coronary angiography: normal epicardial arteries → obstructive CAD excluded.	MINOCA diagnosed (inflammatory / microvascular / vasospastic).
November 2014	Patient agreed to start steroid-sparing maintenance.	–	Azathioprine 2 mg/kg/day (100 mg/day) added.
**Phase 5—Stabilization & treatment discontinuation (2015–2017)**			
July 2015	Neurological improvement, respiratory stabilization, no further cardiac events.	–	Prednisolone taper completed.
2016–2017	Cardiology, pulmonology, and rheumatology follow-up: sustained clinical stability; no ischemic symptoms; stable moderate obstructive airway disease.	Normal TTE and stress test (2017).	Azathioprine reduced to 50 mg (May 2016), then self-discontinued (May 2017).
**Phase 6—Drug-free remission & loss to follow-up (2017–2025)**			
March 2019	Last rheumatology review—clinically stable; subsequently lost to follow-up.	–	Off all immunosuppression.
2019–2025	No reported relapse; mild intermittent distal sensory symptoms. Follow-up continued with other medical specialties, without regular rheumatology follow-up.	No evidence of recurrent systemic disease documented during available follow-up.	Off all immunosuppressive therapy.
**Phase 7—Long-term reassessment (2025–2026)**			
September 2025	Re-presented to rheumatology clinic after 6 years; clinical remission, excellent functional status, minimal symptoms.	Eosinophils 9.2% (0.33 × 10^9^/L); normal inflammatory markers; negative autoimmune tests (ANA, PR3-ANCA and MPO-ANCA, RF). Normal CXR and TTE. Nerve conduction studies: only mild chronic residual axonal changes without evidence of active or progressive neuropathy.	Continued observation off immunosuppression.
October 2026	Telemedicine review—stable condition.	–	–

Abbreviations: AFB, acid-fast bacilli; ANA, antinuclear antibody; ANCA, antineutrophil cytoplasmic antibody; APLA, antiphospholipid antibody; CAD, coronary artery disease; CRP, C-reactive protein; CT, computed tomography; CXR, chest radiograph; EGPA, eosinophilic granulomatosis with polyangiitis; EHL, extensor hallucis longus; ESR, erythrocyte sedimentation rate; IgE, immunoglobulin E; LA, lupus anticoagulant; LVEF, left ventricular ejection fraction; MINOCA, myocardial infarction with non-obstructive coronary arteries; NCS, nerve conduction study; NSTEMI, non-ST-elevation myocardial infarction; PCI, percutaneous coronary intervention; RF, rheumatoid factor; RLL, right lower lobe; SPECT, single-photon emission computed tomography; TB, tuberculosis; TTE, transthoracic echocardiography; β2-GPI, beta-2 glycoprotein I

## Search strategy, literature review and quality assessment

**Fig. 2:**
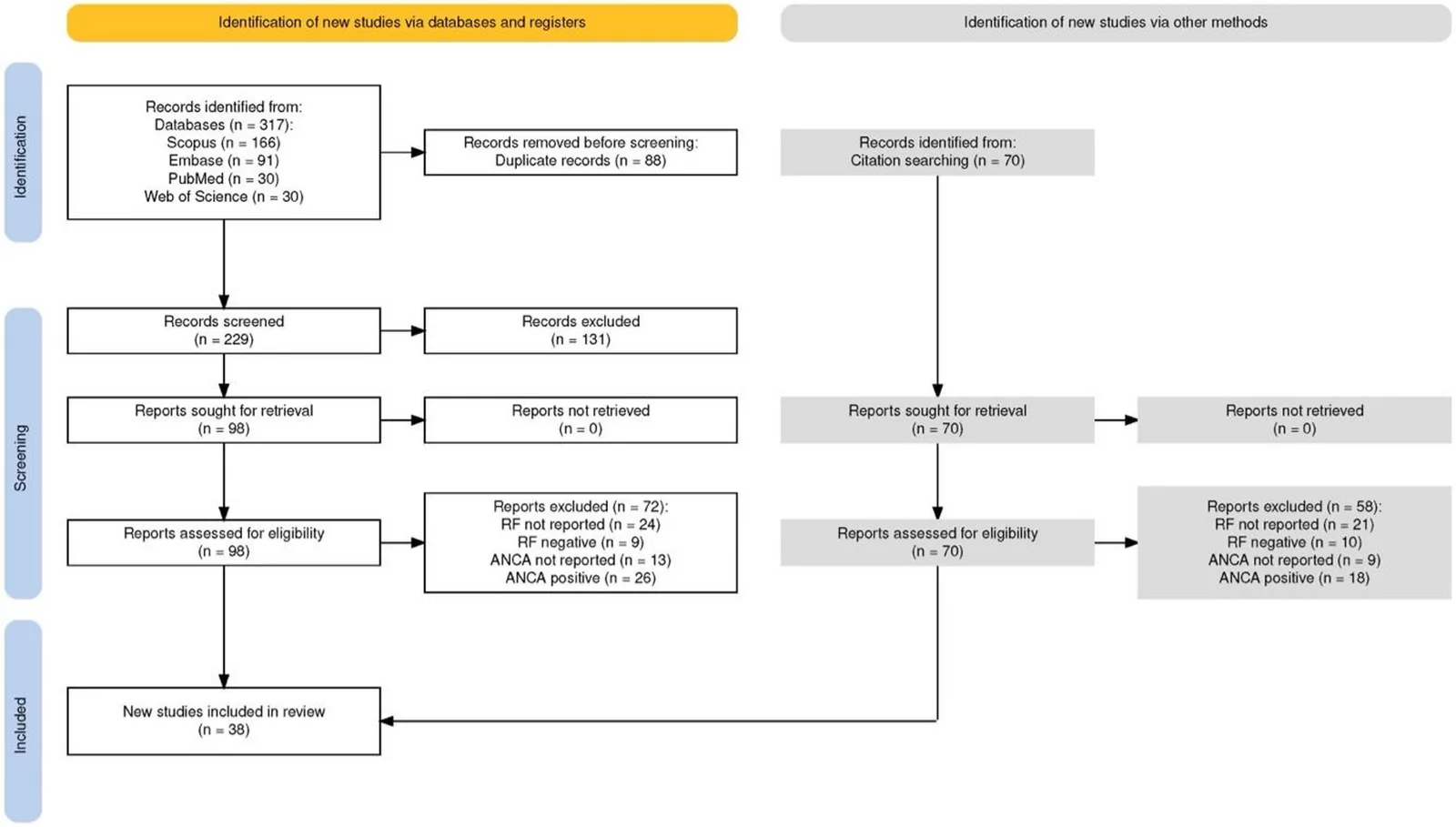
PRISMA search flowchart (last search: 15 August 2026). Abbreviations: MEDLINE, Medical Literature Analysis and Retrieval System Online; PRISMA, Preferred Reporting Items for Systematic Reviews and Meta-Analyses

A case-based review was conducted following the CAse-bAsed REview sTandards (CABARET), with a systematic search of PubMed/MEDLINE, Scopus, Embase, and Web of Science from inception to 15 August 2026. Detailed study-selection methods and the full search strategy are provided in the Supplementary Methods and Supplementary Table S1, respectively. The PRISMA flowchart is shown in Fig. 2. Methodological quality was assessed using the Murad et al. tool [8], with results presented in Supplementary Table S2.

## Discussion

This case illustrates the diagnostic challenge of ANCA-negative EGPA with a gradually evolving, multisystem presentation. Poorly controlled asthma, chronic rhinosinusitis, progressive eosinophilia, a MINOCA-like presentation, and later mononeuritis multiplex initially appeared to be unrelated problems. The positive RF added further diagnostic uncertainty by raising concern for CTD. Considered together, however, these findings formed a coherent pattern of eosinophilic and vasculitic disease. Because no single test confirms EGPA, diagnosis depends on integrating clinical, laboratory, imaging, and, when available, histopathological findings [5].

ANCA negativity does not exclude EGPA, as only approximately 30–40% of patients are ANCA-positive. Current evidence supports partially distinct but overlapping phenotypes: ANCA-positive disease more often exhibits vasculitic features such as glomerulonephritis, peripheral neuropathy, and purpura, whereas ANCA-negative disease is more frequently associated with pulmonary infiltrates and cardiac involvement related to eosinophilic tissue injury [9, 10]. The combination of asthma, sinonasal disease, progressive eosinophilia, cardiac involvement, and neuropathy therefore remained highly compatible with EGPA despite repeatedly negative ANCA testing in our patient.

Cardiac involvement is an important and potentially underrecognized manifestation of EGPA. It may present as myocarditis or an acute coronary syndrome–like illness caused by eosinophilic myocardial injury, coronary vasospasm, thrombosis or vasculitis, and microvascular injury. Moreover, cardiac disease may be present despite normal initial biomarkers, ECG, or echocardiographic findings and carries substantial prognostic significance [9, 11]. In our patient, chest pain, troponin elevation, regional wall-motion abnormality, and reversible perfusion defects occurred without traditional CV risk factors or obstructive epicardial coronary disease. In the context of asthma, chronic rhinosinusitis with nasal polyposis, eosinophilia, and elevated IgE, this cardiac presentation should raise suspicion for EGPA rather than being regarded as an isolated CV event.

Peripheral neuropathy in EGPA typically results from ischemic axonal injury due to small-vessel vasculitis and may initially involve one nerve territory before progressing to a multifocal pattern. MPO-ANCA–mediated neutrophil endothelial injury is more prominent in ANCA-positive disease, whereas eosinophilic cytotoxicity and vascular occlusion may play a greater role in ANCA-negative disease [12]. Suspected neuropathy should therefore prompt nerve conduction studies. Nerve biopsy may support the diagnosis in selected cases, although its sensitivity for definite vasculitis is approximately 50–60% [5, 9, 12].

RF positivity represented a potential diagnostic distraction. RF is nonspecific and has been reported in 11.1%–36.7% of patients with EGPA [3, 13]. A recent multicenter observational study reported high-titer RF in 36% of patients, associated with mononeuritis multiplex, purpura, higher CRP levels at diagnosis, and earlier relapse possibly reflecting enhanced B-cell activation and autoantibody production rather than RA [14]. Similarly, Inamo et al. stratified 16 patients at a median RF of 75 IU/mL; all RF-high patients were MPO-ANCA negative, with marked eosinophilia and gastrointestinal involvement, whereas cardiac involvement clustered in the RF-low group, potentially related to interleukin-5-driven B-cell activation [15]. Our patient shared this RF-high/MPO-ANCA-negative profile but had no synovitis and was anti-CCP negative, arguing against RA. These findings reinforce that RF positivity, even at high titers, is nonspecific and should not dissuade clinicians from considering EGPA when the overall clinical phenotype is compatible.

The multisystem presentation prompted consideration of several mimics. Antiphospholipid syndrome was unlikely because antiphospholipid antibodies were transient and no definite thrombotic event occurred. Infection, malignancy, drug-induced eosinophilia, and hypereosinophilic syndrome were also considered; however, adult-onset asthma, chronic rhinosinusitis with nasal polyposis, progressive eosinophilia, and subsequent mononeuritis multiplex favored EGPA. Ultimately, diagnosis depended on recognizing the longitudinal multisystem pattern rather than any single abnormality.

Despite a baseline Five-Factor Score of 0, cardiac and neurological involvement warranted treatment with prednisolone followed by azathioprine. After discontinuing therapy in 2017, she remained off immunosuppressive therapy for more than nine years without reported relapse. Although continuous rheumatology follow-up was not available throughout this period, she continued to receive medical care from other specialties, including family medicine and pulmonology, and later rheumatology reassessment confirmed continued clinical stability, with only residual neurological sequelae. This prolonged treatment-free remission is notable because, in patients with EGPA without poor prognostic factors, adding azathioprine to glucocorticoids did not improve remission rates, reduce relapse risk, or provide a glucocorticoid-sparing benefit [16], and sustained treatment-free remission appears uncommon in EGPA [17, 18]. The prognostic significance of eosinophil counts remains uncertain [14, 18–20].

Interestingly, our patient was homozygous for the HLA-DRB1*03-DQB1*02 (HLA-DQ2) haplotype, which is commonly associated with HLA-DRB3 (DR52), whereas HLA-DRB4 (DR53) is typically associated with HLA-DRB1*04, *07, and *09 [21], although neither DRB3 nor DRB4 was directly tested. Although earlier candidate-gene studies implicated HLA-DRB4 in EGPA susceptibility, subsequent genome-wide studies indicate that HLA associations are predominantly observed in MPO-ANCA-positive EGPA, whereas ANCA-negative disease has a distinct genetic architecture without significant associations with imputed classical HLA alleles [22–24]. Collectively, these findings highlight the genetic heterogeneity of EGPA and suggest that HLA-related susceptibility may differ according to ANCA status. These associations reflect population-level susceptibility effects and should not be interpreted as diagnostic markers at the individual patient level.

The literature review identified 38 previously published cases of ANCA-negative EGPA with RF positivity, including two patients with pre-existing seropositive RA (Table 2). Compared with the 36 published ANCA-negative, RF-positive EGPA cases without pre-existing RA (Table 3), our patient was younger (42 vs. median 53 years) and had a substantially lower peak absolute eosinophil count (2.3 vs. 9.4 × 10⁹/L). Peripheral neuropathy and cardiac involvement were reported in 72.2% and 47.2% of published cases, respectively, with both occurring in 25.0%; both reported deaths were cardiac. These frequencies reflect manifestations reported in a selected set of published cases and should not be interpreted as estimates of their prevalence in ANCA-negative, RF-positive EGPA. Among the 12 cases with sufficient data to standardize RF relative to the assay upper limit of normal (ULN), 75% had RF levels > 3 × ULN, consistent with the markedly elevated RF in our patient (16.9 × ULN). Reporting was frequently incomplete: both MPO- and PR3-ANCA results were documented in only 42% of cases, while anti-CCP status was reported in only four cases (11%), all of which were negative. No included published case explicitly reported prolonged treatment-free remission; however, follow-up duration and treatment status were incompletely reported in several cases, limiting comparisons with our patient.

**Table 2:** Published cases of ANCA-negative, rheumatoid factor–positive eosinophilic granulomatosis with polyangiitis, with the present case

Case, country, year (age, sex)	Organ involvement	WBC; eosinophils ×10⁹/L (%)	CRP; ESR	IgE	ANCA	RF	Biopsy	Treatment	Outcome and follow-up
**Published cases without pre-existing rheumatoid arthritis (*n* = 36), in chronological order**									
1. De Vlam, Belgium, 1995 (58, F) [25]	A, E, L, P, M	15.7; 8.2ᵃ (52%)	10.5; 20	Normal	Negativeᵇ	Positive, value NR	Muscle: eosinophilic vasculitis	Prednisolone 60 mg/d, tapered	Remission at 1 wk; duration NR
2. Lau, UK, 2004 (31, F) [26]	A, L, C, S	NR; 20.0 (%NR)	72; 50	NR	Negativeᵇ	Titer 1:80	Skin: eosinophilic vasculitis	IV MP 500 mg ×2 → prednisolone 40 mg/d; AZA	Pericardial effusion and thrombi resolved at 6 wk
3. Sakamoto, Japan, 2005 (36, F) [27]	A, P, N, G, S	24.8; 9.5ᵃ (38.4%)	12; NR	1,000	Negative MPO	Positive, value NR	–	Endovascular occlusion of vertebral artery; prednisone	Discharged day 52, no neurological sequelae
4. Ferenczi, USA, 2007 (53, M) [28]	A, E, L, P, S, G, R	24.0; 2.0 (8.3%ᵃ)	64.8; 111	501	Negative cANCA, pANCA	92 IU/mL	Skin: leukocytoclastic vasculitis. Sural nerve: vasculitis. Colon: eosinophilic vasculitis	Prednisone 60 mg/d + CYC 150 mg/d; IV MP 1 g ×3; warfarin; AZA 150 mg/d	Digital gangrene & asthma stabilized; neuropathy & muscle strength mildly improved
5. Shimauchi, Japan, 2007 (53, F) [29]	A, P, S	12.6; 7.2 (57%ᵃ)	NR; 82	1,253	Negativeᵇ	425 IU/Lᶜ	–	Prednisolone 40 mg/d	Resolved at 1 mo; RF fell to 35.3
6. Skrapari, Greece, 2008 (50, F) [30]	A, E, N, P, O	19.0; 3.8 (20%)	46.5; 104	NR	Negativeᵇ	2,010 IU/mL	Sural nerve: eosinophilic vasculitis	IV MP 1 g ×3; IV CYC 800 mg/4 wk; LMWH	Systemic features resolved; vision minimally improved
7. Setoguchi, Japan, 2009 (60, M) [31]	A, C, P	21.4; NR	NR; NR	1,450	Negativeᵇ	230 IU/mL	Autopsy, myocardium: small- and medium-artery vasculitis	Nitrates, nicorandil, morphine	Died—cardiac arrest
8. Taki, Japan, 2010 (39, F) [32]	A, P, S	15.9; 10.6 (66.5%)	3; 75	1,200	Negative MPO, PR3	305 U/mL	Skin and muscle: necrotizing eosinophilic vasculitis	Pranlukast stopped; prednisolone 40 mg/d (1 mg/kg)	Improved at 2 mo; RF fell to 12
9. Horai, Japan, 2011 (58, F) [33]	A, P, G	20.6; 13.2ᵃ (63.9%)	9.8; 35	2,982	Negative MPO, PR3	43 U/mL	Sural nerve: eosinophil, plasma cell and lymphocyte infiltration	GC pulse + CYC pulse → prednisolone 50 mg/d	Significant improvement; prednisolone tapered without relapse
10. Suganuma, Japan, 2011 (57, M) [34]	A, E, C, N, P	20.1; 12.7 (63.2%)	40; NR	NR	Negative MPO, cANCA	711.6 IU/mL	–	Pericardiocentesis; IV MP 1 g ×3 → prednisolone 50 mg/d	Remission on prednisolone 3 mg/d at 1 y; mild gaze palsy
11. Kobayashi, Japan, 2011 (51, F) [35]	A, L, C, P, R	18.2; 10.5 (57.8%)	112.9; NR	NR	Negative MPO, cANCA	30.8 IU/mL	–	IV MP pulse → prednisolone 40 mg/d; IVIG; IV CYC	Controlled on methylprednisolone 12 mg/d + AZA 50 mg/d, able to maneuver wheelchair independently
12. Levine, USA, 2012 (85, F) [36]	A, C, N, P, M, S	16.3; 8.5ᵃ (52%)	72.9; 34	3,385	Negative MPO, PR3	46.4 IU/mL	Right ventricle: eosinophilic myocarditis. Marrow: reactive hypereosinophilia	IV MP 1 g ×3 → prednisone 40 mg/d	Initial improvement in eosinophilia and wrist drop Died—cardiac arrest, day 13
13. Paroli, Italy, 2012 (46, F) [37]	A, L, C, P, N, S	13.0; 8.6 (66%ᵃ)	22.3; 65	324	Negative cANCA, pANCAᵇ	126 IU/mL	Breast skin: periductal necrotizing granulomas, eosinophilic vasculitis	IV MP 1 g ×3 → prednisolone 50 mg/d + CYC 100 mg/d; warfarin	Asthma and neurological deficits improved
14. Harada, Japan, 2012 (56, F) [38]	A, P, G	14.5; 0.8ᵃ (5.6%)ᵈ	129; 70	419	Negative ANCA, MPO	104.9 IU/mL	Liver: hepatic-artery fibrinoid necrosis, portal eosinophilia	IV MP 1 g ×3 → prednisolone 1 mg/kg/d	Fever, eosinophilia and CRP improved; neuropathy resolved
15. Suzuki, Japan, 2014 (42, F) [39]	A, E, C, S	NR; 1.1 (13.5%)	NR; NR	791	Negative MPO, PR3	Positive, low titer, value NR	–	Betamethasone 4 mg/d; diltiazem, isosorbide mononitrate	Eosiniphilia resolved Angina-free with normal angiography at 3 mo
16. Parent, Canada, 2014 (74, M) [40]	A, E, L, M	18.5; 5.1 (27%ᵃ)	173.7; NR	NR	Negative MPO, cANCA	77 U/mL	Muscle: eosinophil-rich necrotizing vasculitis, inflammatory myopathy	Prednisone 80 mg/d + AZA 50 mg/d	Near-resolution at 6 wk; relapse on rapid GC taper
17. Parperis, USA, 2016 (27, F) [41]	P, S, G, R	19.9; 5.6ᵃ (28%)	NR; NR	545	Negative ANCA, MPO, PR3	63 IU/mL	Skin: neutrophilic granulomatous dermatitis, medium-vessel vasculitis	IV MP 1 g/d ×3, then further IV MP	Skin resolved within days; foot drop improved gradually
18. Braunberger, USA, 2016 (72, M) [42]	A, P, S	NR; normal (on GC)	100; 97	NR	Negative pANCA, cANCAᵇ	> 600 IU/mL	Lung: EGPA	IV CYC 1,500 mg; IV MP 250 mg q6h ×72 h → prednisone 60 mg/d; pentoxifylline;	Progressed to multi-toe dry gangrene
19. Alarfaj, Saudi Arabia, 2018 (35, M) [43]	A, E, C, M	17.0; 7.0 (41%ᵃ)	410; 30	1,777	Negative ANCAᵇ	166ᵉ	Endobronchial: eosinophilic vasculitis	IV MP 1 g ×3; CYC ×6 monthly; rituximab; MTX weekly	LV outflow tract mass resolved; no recurrence at 18 mo
20. Civelli, USA, 2020 (62, M) [44]	A, E, C, N, G, R, S	19.9→28.4; 9.7 (48.9%) →13.0 (46%)	16; 23	NR	Negative MPO, PR3	Positive, value NR	Endomyocardium: eosinophilic myocarditis	IV MP 1 g + IV CYC; then CYC and prednisone 5 mg/d for 1 y, then maintained on prednisone	LVEF normalized; minor residual right-sided weakness
21. Ueki, Japan, 2020 (55, M) [45]	A, E, P	25.4; 14.3 (52.6%)	7; NR	NR	Negative MPO	690 IU/mL	–	IV MP 1 g ×4 → prednisolone 60 mg/d; MTX 6 mg/wk; IVIG	Remission > 9 mo on prednisolone 5 mg/d
22. Ueki, Japan, 2020 (53, F) [45]	A, E, L, C, P, G, S, M	27.7; 20.7 (74.6%)	9.4; NR	993	Negative MPO	80 IU/mL	BAL: 74.6% eosinophils	Prednisolone 50 mg/d; IV CYC + IVIG; AZA; cyclosporine; MTX; mepolizumab 300 mg/mo	Two relapses; gradual improvement at 8 mo on mepolizumab
23. Sada, Japan, 2021 (62, F) [46]	A, L, P, N, S	43.4; 36.9 (85%ᵃ)	13.1; NR	NR	Negative MPO, PR3	72 IU/mL	Skin: leukocytoclastic vasculitis with eosinophils	Prednisolone 40 mg/d; mepolizumab 300 mg SC monthly	Remission on prednisolone 5 mg/d
24. Kurihara, Japan, 2021 (66, F) [47]	A, C, P	35.8; 30.6ᵃ (85.5%)	NR; NR	1,862	Negative MPO, PR3	1,101 IU/mL	Endomyocardium: eosinophilic myocarditis. Sural nerve: ischemic vasculitic neuropathy	IV MP 1 g/d ×3, two courses → prednisolone 40 mg/d	Numbness almost resolved by discharge
25. Huckenpahler, USA, 2022 (69, M) [48]	A, E, C, N, R, M ᵍ	26.1; 11.8 (45%)	60.4; NR	NR	Negative MPO, PR3	25 IU/mL	Kidney: pauci-immune crescentic GN, eosinophil-rich interstitial inflammation	IV MP 1 g/d ×3 → prednisone 1 mg/kg/d; rituximab 1 g	Strength returned to baseline; No further SVT; eosinophils and creatinine improved at 6 mo
26. Ueno, Japan, 2022 (74, M) [49]	A, P, G, R, S	38.7; 24.8ᵃ (64%)	127.3; 78	3,393	Negative MPO, PR3	181 U/mL	Skin: leukocytoclastic vasculitis. Stomach and duodenum: eosinophilia	Tapering high-dose GC over 23 mo; mepolizumab 300 mg SC monthly	BVAS 0; remission to 6 mo after GC withdrawal
27. Shanker, UK, 2022 (29, M) [50]	A, E, L, P, S, R, M	9.8; 0.3ᵃ (3.1%)	NR; NR	195	Negative ANCA, MPO	793 IU/mL	Skin: leukocytoclastic vasculitis	Prednisolone 60 mg/d tapered over 8 wk; MMF to 2 g/d	All symptoms resolved; normal counts and IgE at follow-up
28. Ma, China, 2022 (32, F) [51]	A, L, P, G, S	15.6; 8.0 (51.1%)	5.4; 30	808	Negative MPO, PR3	176 IU/mL	–	Prednisone 30 mg/d + CYC 50 mg/d; betamethasone 7 mg IM; stellate ganglion block ×2 courses	Remission; prednisone tapered to 10 mg/d
29. Yamamoto, Japan, 2022 (72, F) [52]	A, C	15.4; 10.4 (67.6%) ᵃ	65; >110	1,840	Negative ANCAᵇ	204 IU/mL	Endomyocardium: eosinophilic infiltrate, fibrinoid necrotizing vasculitis	IV MP 1 g/d ×3 → prednisolone 1 mg/kg/d, tapered	Remission by day 56; relapse day 75; stable on 4 mg/d at 1 y
30. Takahashi, Japan, 2024 (78, F) [53]	A, E, C, P, N, G	10.8; 5.0 (46.2%)	26.3; 54	1,956	Negative MPO, PR3	146.3 IU/mL	Endomyocardium: subendocardial eosinophilic inflammation	Heparin → warfarin; heart-failure therapy; IV MP 1 g/d ×3 → prednisolone 1 mg/kg/d	LV thrombus resolved by day 11; transferred for rehabilitation
31. Matsuda, Japan, 2025 (43, F) [54]	A, L, P, S	4.6; 3.1ᵃ (67.3%)	NR; NR	5,292	Negative ANCAᵇ	Positive, value NR	Skin: eosinophilic and neutrophilic infiltration	Systemic GC; mepolizumab SC monthly	Photosensitivity resolved without hyperpigmentation
32. Araya, Japan, 2025 (18, F) [55]	A, E, L, C, M, G	27.6; 17.9 (65%)	33.8; NR	4,125	Negative MPO, PR3	65 U/mL	Lung, stomach, duodenum, sinus and marrow: eosinophilia	Prednisone for 68 mo; mepolizumab 300 mg q4wk from mo 22, mepolizumab monotherapy for 15 mo	Sustained remission beyond 5 y
33. Vasandani, USA, 2025 (43, F) [56]	A, E, L, C, P, N, S, G	22.1; 14.7 (66%)ᵃ	67; NR	NR	Negative ANCA, MPO	417 IU/mL	Marrow: eosinophilia	IV MP 500 mg twice daily ×3 d → prednisone taper; IV CYC ×6; AZA maintenance	Remission at 4 mo
34. Alajlouni, Canada, 2025 (33, F) [57]	A, E, C	13.4; 6.7 (50%)ᵃ	High; high	920	Negative ANCAᵇ	47 U/mL	–	Stellate ganglion block; IV GC pulse ×5 d → prednisone 1 mg/kg/d + IV CYC ×3 mo; ICD and AZA maintenance; mepolizumab added for asthma relapse, allowing steroid discontinuation	Symptom-free for 1.5 y
35. Tang, China, 2025 (40, F) [58]	A, E, L, P	19.6; 5.3ᵃ (27.1%)	278; normal	705	Negative MPO, PR3	24.8 IU/mL	Marrow and transbronchial: eosinophilia	IV MP 40→500 mg/d; IVIG; CYC 0.6 g q2wk; etanercept 50 mg SC weekly ×3 mo; then MTX	Near-full recovery at 1 y; no recurrence at 2 y
36. Nakagawa, Japan, 2025 (31, F) [59]	A, L, S, M, G	12.8; 8.9 (69.8%)ᵃ	6.7; 14	231	Negative MPO, PR3	178 IU/mL	Skin to muscle: eosinophilic vasculitis	Prednisolone 25 mg/d (0.5 mg/kg/d)	Symptoms improved, eosinophils and CK normalized at 1 wk
**Cases with pre-existing seropositive rheumatoid arthritis (*n* = 2)—presented separately; RF positivity is attributable to rheumatoid arthritis**									
37. Asano, Japan, 2020 (59, F) [60]	A, P, S	10.6; 3.3ᵃ (31.5%)	80.3; NR	1,042	Negative MPO, PR3	436.2 IU/mL	Skin: concurrent IgA vasculitis and EGPA	Prednisolone 50 mg/d; IVIG 20 g/d ×5	Purpura resolved; maintained on prednisolone 10 mg/d + AZA
38. Imai, Japan, 2022 (83, F) [61]	A, E, C, G	16.6; 4.5ᵃ (27.4%)ʰ	180.1; NR	2,200	Negative MPO, PR3	401 IU/mL	Stomach and duodenum: eosinophilic gastroenteritis	Prednisolone 45 mg/d; IV CYC 500 mg q2wk	Remission > 1 y on prednisolone 4 mg/d + AZA 50 mg/d
**Present case**									
39. Alghaithi, UAE, 2026 (42, F)	A, E, C, P	6.7; 2.3 (32.3%)	55.4; 11	1,040	Negative MPO, PR3	322 IU/mL	Declined (nerve)	Prednisolone 45 mg/d (1 mg/kg); AZA 100 → 50 mg/d, self-discontinued 2017; pregabalin	Treatment-free remission for > 9 y without reported relapse; mild residual sensory symptoms

Organ involvement: A, asthma; C, cardiac; E, ear, nose and throat (sinusitis, rhinitis or nasal polyposis); G, gastrointestinal or hepatobiliary; L, lung (infiltrates, hemoptysis or alveolar eosinophilia); M, musculoskeletal (myositis or arthritis); N, central nervous system; O, ocular; P, peripheral nerve; R, renal; S, skin

Laboratory values: White cell and eosinophil counts are expressed throughout as ×10⁹/L; values originally published as /mm³, /µL or ×10³/µL were converted by dividing by 1,000, and values already in ×10⁹/L are unchanged. CRP is given in mg/L, ESR in mm/h and IgE in IU/mL. Reference ranges are shown in parentheses only where the source reported them

*Abbreviations*: ANCA, antineutrophil cytoplasmic antibody; AZA, azathioprine; BAL, bronchoalveolar lavage; BVAS, Birmingham Vasculitis Activity Score; CK, creatine kinase; CLIA, chemiluminescence immunoassay; CRP, C-reactive protein; CYC, cyclophosphamide; EGPA, eosinophilic granulomatosis with polyangiitis; ELISA, enzyme-linked immunosorbent assay; ESR, erythrocyte sedimentation rate; F, female; GC, glucocorticoid; GN, glomerulonephritis; ICD, implantable cardioverter-defibrillator; IgE, immunoglobulin E; IV, intravenous; IVIG, intravenous immunoglobulin; LMWH, low-molecular-weight heparin; LV, left ventricular; LVEF, left ventricular ejection fraction; M, male; MMF, mycophenolate mofetil; MP, methylprednisolone; MPO, myeloperoxidase; MTX, methotrexate; NR, not reported; PR3, proteinase 3; RF, rheumatoid factor; SC, subcutaneous; SVT, supraventricular tachycardia; WBC, white blood cell

^a^Calculated: the absolute eosinophil count was derived from the reported white cell count and eosinophil percentage, or the percentage from the two counts, where the source reported only one

^b^ANCA reported as negative without the antigen specificity (MPO, PR3) being stated

^c^Published as 425 IU/L; interpreted as IU/mL, since the stated cutoff (< 20) and the reported post-treatment value both correspond to the IU/mL convention

^d^Described in the source as a normal eosinophil count, although the derived absolute count (0.8 × 10⁹/L) exceeds the conventional upper limit of 0.5 × 10⁹/L

^e^Reported without units

^f^Sibling of the preceding case (familial EGPA)

^g^Authors reported ‘neuropathy’ as an ACR criterion, but the described right-arm weakness/numbness coincided with cerebral infarction; no independent evidence of peripheral neuropathy or mononeuritis multiplex was provided

^h^The source reports an absolute eosinophil count of 18,550/µL alongside a white cell count of 16,600/µL and an eosinophil fraction of 27.4%, which are mutually incompatible; the count shown here is derived from the white cell count

**Table 3:** Summary of 36 published cases of ANCA-negative, rheumatoid factor–positive EGPA, compared with the present case

Characteristic	Published cases (*n* = 36)	Present case
**Demographics and geographic distribution**		
Age at presentation, years—median (range)	53 (18–85)	42
Female sex	25 (69%)	Female
Country of report	Japan 19 (53%), USA 7 (19%), other 10 (28%)^a^	United Arab Emirates
**Clinical features**—n / cases in which the feature was addressed (%)^b^		
Asthma	35/36 (97.2%)	Yes
Sinusitis (acute or chronic)	16/19 (84.2%)	Yes
Nasal polyps	7/8 (87.5%)	Yes
Cardiac involvement	17/36 (47.2%)	Yes (MINOCA)
Peripheral neuropathy	26/36 (72.2%)	Yes (mononeuritis multiplex)
Both cardiac and peripheral nerve involvement	9/36 (25.0%)	Yes
Skin involvement	19/19 (100%)	No
Gastrointestinal or hepatobiliary involvement	13/13 (100%)	No
Hematuria	5/10 (50.0%)	No
**Laboratory findings**—median (range); n reporting the parameter / 36		
White cell count, ×10⁹/L	19.0 (4.6–43.4); 33/36	6.7
Absolute eosinophil count, ×10⁹/L	9.4 (1.1–36.9); 23/36	2.3
Eosinophils, % of white cells	52.0 (3.1–85.5); 25/36	32.3
CRP, mg/L	43.2 (3.0–410.0); 28/36	55.4
ESR, mm/h	60 (14–111); 18/36^c^	11
IgE, IU/mL	1,100 (195–5,292); 24/36	1,040
**ANCA testing**—completeness of reporting; n (%) of 36		
MPO- and PR3-specific antibodies both reported negative	15 (42%)	Yes (ELISA, then CLIA)
Single antigen-specific assay only (MPO) reported negative	9 (25%)	–
Immunofluorescence only (cANCA / pANCA) reported negative, antigen specificity not reported	3 (8%)	–
Reported as “ANCA negative” with no assay or subtype stated	9 (25%)	–
**Rheumatoid factor and anti-CCP**		
Quantitative RF value with units reported	29 (81%)^d^	Yes
RF, IU/mL—median (range)	146 (25–2,010)^e^	322
RF as a multiple of the assay upper limit of normal—median (range)	11.2 (1.8–100.5); 12/36	16.9
RF > 3 × upper limit of normal	9/12 (75%)^f^	Yes
Anti-CCP status reported	4 (11%)	Yes
Anti-CCP negative, of those reported	4/4 (100%)	Negative
**Treatment**—n (%) of 36		
Glucocorticoids	35 (97%)	Yes (prednisolone 1 mg/kg/d)
Cyclophosphamide	13 (36%)	No (declined)
Azathioprine	7 (19%)	Yes
Mepolizumab	6 (17%)	No
Rituximab	2 (6%)	No (declined)
**Outcome**		
Death during reported follow-up	2 (6%)	No
Duration of treatment-free remission reported	0 (0%)	∼ 9.5 years

Presented separately: two further ANCA-negative, RF-positive EGPA cases arose in patients with pre-existing seropositive rheumatoid arthritis (Asano et al., Japan, 2020, RF 436.2 IU/mL; Imai et al., Japan, 2022, RF 401 IU/mL). Because RF positivity in these patients is attributable to rheumatoid arthritis, they are excluded from every proportion above

Data are n (%) of 36 unless otherwise stated. For laboratory parameters, the denominator is the number of cases reporting that parameter and is shown in the cell. White cell and eosinophil counts published as /mm³, /µL or ×10³/µL were converted to ×10⁹/L by dividing by 1,000

^a^ Belgium (1), Canada (2), China (2), Greece (1), Italy (1), Saudi Arabia (1) and the United Kingdom (2)

^b^ The denominator represents the number of cases in which the feature was addressed, either reported as present or explicitly reported as absent, rather than all 36 cases. Cases in which the feature was not mentioned were excluded, as non-reporting does not establish absence. A denominator of 36 indicates that the feature was addressed in all cases. For sinusitis, 19 cases provided sufficient information, of which 16 had sinusitis. For skin and gastrointestinal involvement, no case explicitly reported absence; therefore, the denominator equals the number of cases in which the feature was present, and the true proportion cannot be estimated

^c^ One value reported as > 110 mm/h was analyzed as 110. Three further cases described CRP or ESR only as “high” or “normal” without a value and are excluded from the denominator

^d^ Of the seven remaining cases, five reported RF as positive without a value, one reported a numeric value without units, and one reported a titer (1:80) only. Rheumatoid factor units differed between reports: IU/mL in 22 cases, U/mL in 6, IU/L in 1 and absent in 1. Values in U/mL were treated as equivalent to IU/mL

^e^ One report expressed rheumatoid factor as 425 IU/L alongside a stated cutoff of < 20; because the cutoff and the reported post-treatment value both correspond to the IU/mL convention, this was interpreted as 425 IU/mL. Excluding it changes the median multiple of the upper limit of normal from 11.2 to 10.5. One value reported as > 600 IU/mL was analyzed as 600 IU/mL. Assays and cutoffs differed across three decades and nine countries; these figures are descriptive and are not a pooled estimate

^f^ Denominator is the 12 cases reporting both a value and the assay reference range

Abbreviations: ANCA, antineutrophil cytoplasmic antibody; anti-CCP, anti-cyclic citrullinated peptide antibody; CLIA, chemiluminescence immunoassay; CRP, C-reactive protein; EGPA, eosinophilic granulomatosis with polyangiitis; ELISA, enzyme-linked immunosorbent assay; ESR, erythrocyte sedimentation rate; IgE, immunoglobulin E; MINOCA, myocardial infarction with non-obstructive coronary arteries; MPO, myeloperoxidase; PR3, proteinase 3; RF, rheumatoid factor

The marked variability and incompleteness of reporting across published cases limited direct comparisons between cases. Based on these gaps, we propose an author-developed reporting checklist for future EGPA case reports to improve the consistency, transparency, and comparability of published data (Table 4). This checklist is intended as a practical reporting aid and has not been formally validated.

**Table 4:** Proposed reporting checklist for future case reports of EGPA

Domain	Recommended reporting
**Part A—general dataset. Recommended reporting for the overall assessment of EGPA.**	
Diagnostic basis	Clinical and/or pathological basis of the diagnosis; state whether classification criteria were fulfilled, and give the score (e.g., 2022 ACR/EULAR criteria for EGPA).
Laboratory investigations *Report every value in SI units with the local reference range, at diagnosis, at remission, and at last follow-up*.	Hematology—Complete blood count with white cell differential, as absolute counts and percentages; serial eosinophil counts.
	Acute-phase response—C-reactive protein and erythrocyte sedimentation rate.
	ANCA—Indirect immunofluorescence pattern together with MPO- and PR3-specific antibodies, with the assay named (e.g., ELISA, CLIA).
	Mimicker screen—Rheumatoid factor (titer or level), anti-CCP, ANA, ENA, complement (C3, C4), cryoglobulins, and antiphospholipid antibodies (anticardiolipin, anti-β2-glycoprotein I, lupus anticoagulant); hepatitis B and C, HIV, syphilis, tuberculosis (IGRA or equivalent), and parasitic infection including *Strongyloides* where geographically relevant; tumor markers.
	Eosinophil biology—Total serum IgE in all cases; allergen-specific IgE (e.g., *Aspergillus fumigatus*); blood IL-5 where available.
	Organ function—Renal and hepatic profile; urinalysis with microscopy.
Treatment	Generic names, doses, routes, and duration of both induction and maintenance therapy.
Follow-up	Total duration of follow-up; disease activity assessment with acute-phase markers and eosinophil counts; timing of and reason for treatment discontinuation; relapse status.
**Part B—organ-directed assessment. Report when the corresponding system is involved or suspected.**	
Cardiac	Cardiac enzymes, electrocardiogram, and transthoracic echocardiography; cardiac MRI and coronary angiography.
Central nervous system	CT head, MRI brain, and MR angiography.
Peripheral nervous system	Nerve conduction studies; nerve biopsy where feasible.
Respiratory	High-resolution CT of the chest, pulmonary function tests, bronchoalveolar lavage, and lung biopsy.
Histopathology	Biopsy of the involved organ (e.g., skin, nerve, stomach, duodenum, endomyocardium, lung) with the findings reported—or an explicit statement of why biopsy was not performed.

Normal acute-phase reactants and a normal blood eosinophil count do not exclude active EGPA, particularly in ANCA-negative, eosinophil-driven disease or after corticosteroid exposure. Where an investigation listed above was not performed, this should be stated explicitly rather than omitted.

ACR, American College of Rheumatology; ANA, antinuclear antibody; ANCA, antineutrophil cytoplasmic antibody; anti-CCP, anti-cyclic citrullinated peptide antibody; CLIA, chemiluminescence immunoassay; CT, computed tomography; EGPA, eosinophilic granulomatosis with polyangiitis; ELISA, enzyme-linked immunosorbent assay; ENA, extractable nuclear antigen; EULAR, European Alliance of Associations for Rheumatology; HIV, human immunodeficiency virus; IGRA, interferon-γ release assay; IgE, immunoglobulin E; IL-5, interleukin-5; MPO, myeloperoxidase; MR, magnetic resonance; MRI, magnetic resonance imaging; PR3, proteinase 3; SI, Système International

## Strengths and limitations

Strengths of our case report include longitudinal documentation of the evolving multisystem presentation, more than nine years off immunosuppressive therapy without reported relapse, and the systematic literature review. Limitations include the absence of histopathological confirmation and cardiac MRI or endomyocardial biopsy, leaving the precise mechanism of the cardiac presentation inferential. In addition, continuous rheumatology follow-up was not available throughout the entire treatment-free interval, although the patient continued to receive medical care from other specialties and later rheumatology reassessment confirmed continued clinical stability. The diagnosis was based on the characteristic longitudinal clinical presentation, progressive eosinophilia, multifocal axonal neuropathy, and exclusion of important mimics. Fulfillment of the 2022 ACR/EULAR classification criteria further supported classification as EGPA but was not used as an independent diagnostic confirmation. Regarding the literature review, its findings should be interpreted in the context of publication bias, selective reporting, substantial heterogeneity and incompleteness of case-level data, and the absence of a comparator group, which precludes determining whether RF positivity identifies a distinct clinical phenotype or has independent diagnostic or prognostic significance in EGPA.

## Conclusion

This case highlights the diagnostic challenge of ANCA-negative EGPA and the potential for RF positivity to distract from the underlying diagnosis. In patients with asthma and eosinophilia, myocardial infarction–like presentations with non-obstructive coronary arteries, particularly when accompanied by sinonasal disease and mononeuritis multiplex, should raise suspicion for eosinophilic and vasculitic disease. Recognition of the evolving multisystem pattern may facilitate earlier diagnosis and treatment. The prolonged period off immunosuppressive therapy without reported relapse represents an unusual long-term outcome but should not be generalized to EGPA more broadly.

## Declarations

### External editing services

External editing services were not used in preparing this manuscript. ChatGPT (OpenAI, version GPT-5.6) was used only for copy editing purposes, such as grammar and clarity, and not for content generation or interpretation.

### Disclaimer

No part of this report has been copied or previously published elsewhere, and its scientific content was not generated by artificial intelligence (AI).
